# Challenges of FGFR2 Testing in Gastric Cancer

**DOI:** 10.3389/or.2023.11790

**Published:** 2023-12-14

**Authors:** Ilya Tsimafeyeu, Grigory Raskin

**Affiliations:** ^1^ Bureau for Cancer Research, New York Office, New York, NY, United States; ^2^ Dr. Berezin Medical Institute, Saint Petersburg, Russia

**Keywords:** gastric cancer, fibroblast growth factor receptor 2, expression, amplification, heterogeneity

## Introduction

In spite of a decrease in the incidence of gastric cancer, the mortality rate from this tumor remains quite high and new approaches to treatment are required [1]. Currently, systemic therapy for metastatic gastric cancer includes chemotherapy, targeted therapy, and immunotherapy. Anti-HER2 and anti-VEGF agents remain the standard of care in the first- and second-line treatments [2]. CLAUDIN 18.2-targeted therapy could be a novel approach to treatment in patients with CLAUDIN 18.2-expressing tumors [3]. Checkpoint inhibitors have demonstrated efficacy in first-line therapy and beyond, especially in patients with PD-L1 expression and MSI-H/dMMR adenocarcinomas.

Fibroblast growth factor receptor 2 (FGFR2) plays an important role in the pathogenesis of various malignant tumors, including gastric cancer. Therefore, it is a prospective alternative target for targeted therapy [4]. To magnify the clinical benefits, patient selection for FGFR2 treatment is based on an evaluation of FGFR2 expression or amplification in the tumor. At this stage, detection problems can arise that lead to erroneous selection of patients. There are several problems and challenges of FGFR2 testing in metastatic gastric adenocarcinoma.

## Challenges of FGFR2 Testing in Metastatic Gastric Adenocarcinoma

Recently, there has been a development of small-molecule tyrosine kinase inhibitors, monoclonal antibodies, as well as allosteric extracellular inhibitors that block FGFR2 [5–7]. The presence of FGFR2 expression or amplification seems to be one of the patient selection factors for targeted therapy of gastric adenocarcinoma. For example, bemarituzumab, an anti-FGFR2 humanized monoclonal antibody, was found to be more effective in patients with FGFR2 expression assessed by immunohistochemistry (HR = 0.52) [6]. Prespecified exploratory analyses in the randomized phase 2 FIGHT study of 155 patients with metastatic gastric cancer showed that progression-free survival (PFS) and overall survival (OS) were better in patients with FGFR2 immunohistochemical expression greater than 5%. However, the authors also found significantly improved OS in intention-to-treat population (HR = 0.58).

In translational clinical studies, gastric cancers with high-level clonal FGFR2 amplification have also been shown to respond better to treatment with selective FGFR tyrosine kinase inhibitors, whereas cancers with low-level amplification did not respond [8, 9].

Therefore, the results of these and other studies indicate that it is necessary to assess expression or amplification prior to initiating FGFR2-targeted therapy and several questions arise as to how to conduct this assessment. Should the primary tumor or metastasis tissue be used for evaluation? Is there tumor heterogeneity? What percentage of FGFR2-expressing cancer cells is needed to make a conclusion about FGFR2-positivity? Finally, the question remains of how well different assays agree on the FGFR2 status of the same patient and whether one test can be substituted by another. Unfortunately, there are no clear answers, however, the upcoming problems with the assessment are obvious.

**FIGURE 1:**
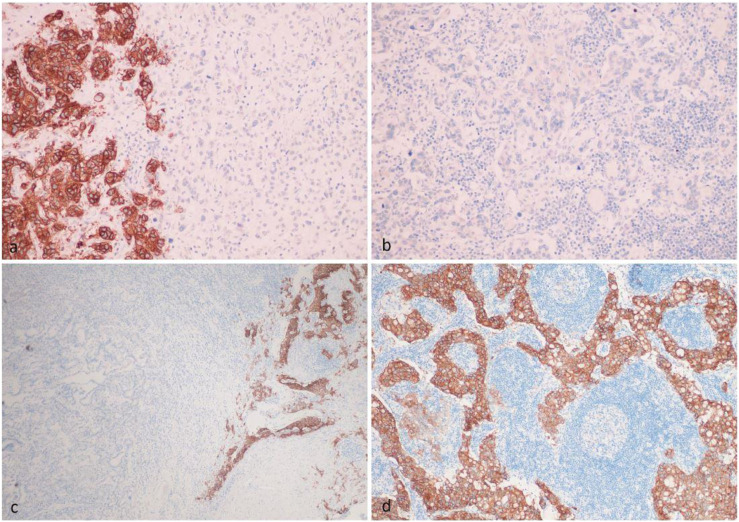
Intratumor heterogeneity of FGFR2 IHC expression in the primary tumor **(A,C)**, which led to the appearance of FGFR2-negative metastasis **(B)** and FGFR2-positive metastasis **(D)** in the same patient.

One of the main problems may be the false-negative selection of patients due to tumor heterogeneity. It is already known that HER2 and PD-L1 heterogeneity encompasses not only interpatient variability (intertumor heterogeneity), but also variations within the same tumor (intratumor heterogeneity) [10–12]. In a retrospective study, we evaluated 109 patients with locally advanced or metastatic gastric adenocarcinoma [13]. Overall, FGFR2 expression was detected in 43% cases and amplification in 8% cases [14]. FGFR2 expression was assessed in the primary tumor as well as in several lymph node metastases from the same patients by immunohistochemistry with three different antibodies (Abcam clone EPR24075-418, R&D clone 98706, Santa Cruz clone C-8). After evaluating the expression in the first 19 patients, further study was carried out only using the Abсam EPR24075-418 assay due to pronounced nuclear staining with other tests. FGFR2 any level expression was detected in 29 (47%) primary tumors and 18 (40%) metastases. However, the level of expression (1+, 2+, 3+) and the percentage of stained cells varied in 4%–22% of cases. Expression (3+) in all tumor cells was detected in only one patient, and heterogeneity of staining was detected in all other cases. A remarkable example of intratumor heterogeneity is the assessment of FGFR2 expression in our patients with stage III gastric cancer. FGFR2 expression was high in one part of the primary tumors, while no expression was detected in another part of the tumors (Figures 1A, C). It was shown that such a situation may lead to node metastasis without expression (Figure 1B), and to lymph node metastasis with strongly expressed FGFR2 (Figure 1D). If such patients are considered to be candidates for FGFR2 targeted therapy, an incorrect decision may be made at the screening stage if a negative part of the tumor is accidently selected during immunohistochemical evaluation. The results of other large studies also demonstrate a high prevalence of FGFR2 heterogeneity in patients with gastric cancer [15–19]. Han et al. included 188 patients and showed that intratumor heterogeneity of FGFR2b protein and FGFR2 mRNA overexpression was observed in 5 of 9 (55.5%) and 18 of 21 (85.7%) cases, respectively [15]. Discordant FGFR2b and FGFR2 expression results between primary and matched metastatic lymph nodes were observed in 5 of 9 (55.5%) and 4 of 14 (28.6%) cases. In a major study (N = 1,974), heterogeneity was present in various primary tumor samples with an increase in *H*-values in their metastatic lymph nodes [16]. More specifically, *H*-scores were 10–61.8 in primary gastric cancers and 130–210 in metastatic lymph nodes, respectively. Seven of 88 (8%) cases showed FGFR2b overexpression in either primary or metastatic gastric cancers; 3 (3%) cases were positive in both the primary and paired metastatic samples; and 4 (5%) cases were positive only in metastatic lymph nodes. In a large central European cohort study (N = 493), less than 1% of tumor cells were stained in 50 cases with strong immunostaining (3+) [17]. No immunostaining (0) of a portion of the tumor was found in 491 (99.6%) tumors. A complete lack of FGFR2 in the entire tumor area was observed in 251 (50.9%) cases. The majority (99.1%) of FGFR2-positive tumors showed a variable combination of staining intensities. More than half (56.6%) of the cases showed even more than two different staining intensities. Collectively, these data show that the expression (combination of intensity of immunostaining and amount of immunopositive tumor areas) of FGFR2 is heterogeneous in gastric cancer.

Interestingly, the heterogeneity may also relate to different isoforms of the receptor. Yashiro et al. enrolled 562 patients [18] and found that all FGFR2IIIc-positive tumors were also positive for FGFR2IIIb in the same tumor, but both were not positive in the same cancer cells. Cases showed heterogeneous expression of both FGFR2IIIb isoform and FGFR2IIIc isoform in a primary tumor. In contrast, most FGFR2-positive tumors were positive for FGFR2IIIb but not FGFR2IIIc.

Evaluation of multiple tumor specimens or multiple biopsies may be considered as an approach to reduce the false negative rate. Ye et al. demonstrated the benefit of multiple biopsy sampling when considering a personalized biomarker strategy [19]. If 3 biopsies were collected from a single patient, the false negative risk for FGFR2 detection was 12.2%. When 6 biopsies were collected, the false negative risk approached 0%. Their study (N = 166) also showed low, medium, and high heterogeneity in 56%, 33%, and 11%, respectively.

Hypothetically, the use of circulating tumor DNA (ctDNA) could improve the results of FGFR2 molecular testing. In a nationwide plasma genomic profiling study GOZILA in Japan, FGFR2 amplification status in paired tissue and plasma samples with advanced gastric cancer was assessed [20]. There was a significantly higher prevalence of FGFR2 amplification in plasma (7.7%) than by tissue analysis alone (2.6%–4.4%). These findings indicated that ctDNA sequencing may identify FGFR2 amplification that cannot be detected by conventional tissue analysis. FIGHT is the first randomized study designed to investigate the efficacy and safety of an FGFR antibody in patients with metastatic gastric cancer using a combination of a blood-based ctDNA next-generation sequencing testing and tumor tissue immunohistochemistry [6]. In the study population, the prevalence of FGFR2 amplification and expression was 4% and 29%, consistent with data reported in previous studies. However, the fact that the addition of bemarituzumab to chemotherapy resulted in promising clinical efficacy in patients with FGFR2b overexpression, regardless of FGFR2 amplification status, supports the selection of patients for future trials of bemarituzumab using immunohistochemistry alone. Similar results were obtained in a Phase 1b study investigating the allosteric extracellular inhibitor alofanib [21]. Immunohistochemical FGFR2 expression proved to be a more clinically relevant than amplification. Other study also showed that fluorescence *in situ* hybridization should not be recommended as a substitute for a FGFR2 immunohistochemistry assay due to the high probability of false negative prediction as a result of intratumor heterogeneity and low Pearson correlation coefficients [13].

The efficacy of checkpoint inhibitors may also correlate with expression of FGFR2, especially in the tumor microenvironment. Ongoing prospective studies may answer this question [22].

## Discussion

Taken together, the results of many studies demonstrate the need to correctly select patients with gastric cancer for FGFR2-targeted therapy. However, the best universal testing option, which would have a high degree of reliability, has not yet been determined. Therefore, evaluation of different testing approaches for each compound appears to be appropriate in clinical and translational studies. Intratumor heterogeneity can become a serious obstacle, as this can reduce accuracy when selecting patients. New principles for pathological evaluation (evaluation of multiple sites in tumors and metastases, etc.) should be explored and standardized.
